# Multimorbidity and care for hypertension, diabetes and HIV among older adults in rural South Africa

**DOI:** 10.2471/BLT.18.217000

**Published:** 2018-10-31

**Authors:** Angela Y Chang, F Xavier Gómez-Olivé, Jennifer Manne-Goehler, Alisha N Wade, Stephen Tollman,, Thomas A Gaziano, Joshua A Salomon

**Affiliations:** a Department of Global Health and Population, Harvard T.H. Chan School of Public Health, 677 Huntington Avenue, Boston, Massachusetts, United States of America (USA).; b MRC/Wits Rural Public Health and Health Transitions Research Unit, School of Public Health, Faculty of Health Sciences, University of the Witwatersrand, Johannesburg, South Africa.; c Division of Infectious Diseases, Massachusetts General Hospital, Boston, USA.; d Department of Cardiovascular Medicine, Harvard Medical School, Boston, USA.; e Department of Medicine, Stanford University School of Medicine, Stanford, USA.

## Abstract

**Objective:**

To examine how multimorbidity might affect progression along the continuum of care among older adults with hypertension, diabetes and human immunodeficiency virus (HIV) infection in rural South Africa.

**Methods:**

We analysed data from 4447 people aged 40 years or older who were enrolled in a longitudinal study in Agincourt sub-district. Household-based interviews were completed between November 2014 and November 2015. For hypertension and diabetes (2813 and 512 people, respectively), we defined concordant conditions as other cardiometabolic conditions, and discordant conditions as mental disorders or HIV infection. For HIV infection (1027 people) we defined any other conditions as discordant. Regression models were fitted to assess the relationship between the type of multimorbidity and progression along the care continuum and the likelihood of patients being in each stage of care for the index condition (four stages from testing to treatment).

**Findings:**

People with hypertension or diabetes plus other cardiometabolic conditions were more like to progress through the care continuum for the index condition than those without cardiometabolic conditions (relative risk, RR: 1.14, 95% confidence interval, CI: 1.09–1.20, and RR: 2.18, 95% CI: 1.52–3.26, respectively). Having discordant comorbidity was associated with greater progression in care for those with hypertension but not diabetes. Those with HIV infection plus cardiometabolic conditions had less progress in the stages of care compared with those without such conditions (RR: 0.86, 95% CI: 0.80–0.92).

**Conclusion:**

Patients with concordant conditions were more likely to progress further along the care continuum, while those with discordant multimorbidity tended not to progress beyond diagnosis.

## Résumé

**Objectif:**

Examiner comment la multimorbidité peut affecter la progression le long du continuum de soins chez les personnes âgées souffrant d'hypertension, de diabète et d'une infection au virus de l’immunodéficience humaine (VIH) dans les zones rurales d'Afrique du Sud.

**Méthodes:**

Nous avons analysé des données provenant de 4447 individus âgés de 40 ans ou plus qui ont participé à une étude longitudinale dans le sous-district d'Agincourt. Des entretiens avec les ménages ont été réalisés entre novembre 2014 et novembre 2015. Dans les cas de l'hypertension et du diabète (2813 et 512 personnes, respectivement), nous avons défini les troubles concordants comme étant d'autres troubles cardiométaboliques, et les troubles discordants comme étant des troubles mentaux ou une infection à VIH. Dans le cas de l'infection à VIH (1027 personnes), nous avons défini tout autre trouble comme étant discordant. Des modèles de régression ont été ajustés pour évaluer le lien entre, d'un côté, le type de multimorbidité et, de l'autre, la progression le long du continuum de soins et la probabilité que les patients suivent chaque étape des soins pour le trouble en question (quatre étapes du dépistage au traitement).

**Résultats:**

Les individus souffrant d'hypertension ou de diabète et d'autres troubles cardiométaboliques étaient davantage susceptibles de progresser le long du continuum de soins pour le trouble en question que ceux qui ne souffraient pas de troubles cardiométaboliques (risque relatif, RR: 1,14, intervalle de confiance, IC, à 95%: 1,09–1,20, et RR: 2,18, IC à 95%: 1,52–3,26, respectivement). La comorbidité discordante a été associée à une progression plus importante le long du continuum de soins dans le cas des individus souffrant d'hypertension, mais non de diabète. La progression le long des étapes de soins était moins importante pour les individus souffrant d'une infection à VIH et de troubles cardiométaboliques que pour ceux non atteints de troubles cardiométaboliques (RR: 0,86, IC à 95%: 0,80–0,92).

**Conclusion:**

Les patients souffrant de troubles concordants étaient davantage susceptibles de mieux progresser le long du continuum de soins, tandis que les patients présentant une multimorbidité discordante avaient tendance à ne pas progresser au-delà du diagnostic.

## Resumen

**Objetivo:**

Examinar cómo la multimorbilidad podría afectar la progresión en la continuidad de la atención entre los adultos de mayor edad con hipertensión, diabetes e infección por el virus de inmunodeficiencia humana (VIH) en las zonas rurales de Sudáfrica.

**Métodos:**

Se analizaron los datos de 4447 personas de 40 años o más inscritas en un estudio longitudinal en el subdistrito de Agincourt. Las entrevistas domésticas se realizaron entre noviembre de 2014 y noviembre de 2015. Para la hipertensión y la diabetes (2813 y 512 personas, respectivamente), se definieron las afecciones concordantes como otras afecciones cardiometabólicas y las afecciones discordantes como trastornos mentales o infección por VIH. Para la infección por VIH (1027 personas) se definió cualquier otra condición como discordante. Se ajustaron los modelos de regresión para evaluar la relación entre el tipo de multimorbilidad y la progresión en la continuidad de la atención y la probabilidad de que los pacientes pasen por cada etapa de atención para la afección en cuestión (cuatro etapas desde la prueba hasta el tratamiento).

**Resultados:**

Las personas con hipertensión o diabetes además de otras afecciones cardiometabólicas tenían más probabilidades de progresar en la continuidad de la atención para la afección en cuestión que las que no tenían afecciones cardiometabólicas (riesgo relativo, RR: 1,14; intervalo de confianza, IC, del 95%: 1,09-1,20; y RR: 2,18, IC del 95%: 1,52-3,26, respectivamente). La comorbilidad discordante se asoció con una mayor progresión en la atención de los pacientes con hipertensión, pero no con diabetes. Aquellos con infección por VIH además de afecciones cardiometabólicas tuvieron un menor progreso en las etapas de atención en comparación con aquellos sin tales afecciones (RR: 0,86, IC del 95%: 0,80-0,92).

**Conclusión:**

Los pacientes con afecciones concordantes eran más propensos a progresar más a lo largo de la continuidad de la atención, mientras que los pacientes con multimorbilidad discordante tendían a no progresar más allá del diagnóstico.

## ملخص

**الغرض:**

دراسة كيفية تأثير تعدد المراضة على التقدم على طول سلسلة الرعاية بين كبار السن الذي يعانون من ارتفاع ضغط الدم والسكري وعدوى فيروس نقص المناعة البشرية (HIV) في المناطق الريفية في جنوب أفريقيا.

**الطريقة:**

قمنا بتحليل البيانات من 4447 شخصًا يبلغون من العمر 40 عامًا أو أكثر، والذين تم تسجيلهم في دراسة طولانية في مقاطعة Agincourt الفرعية. تم الانتهاء من المقابلات المنزلية بين نوفمبر/تشرين ثاني 2014 ونوفمبر/تشرين ثاني 2015. بالنسبة لارتفاع ضغط الدم ومرض السكري (تمت مقابلة 2813 شخصاً و512 شخصا، على الترتيب)، حددنا حالات متطابقة مثل حالات الأيض القلبي الأخرى، وحالات متضاربة مثل الاضطرابات العقلية أو عدوى فيروس نقص المناعة البشرية (HIV). بالنسبة لعدوى فيروس نقص المناعة البشرية (1027 شخصاً) قمنا بتعريف أية حالات أخرى متضاربة. تم توظيف نماذج الانحدار لتقييم العلاقة بين نوع تعدد المراضة والتقدم على طول سلسلة الرعاية، واحتمال وجود المرضى في كل مرحلة من مراحل الرعاية لحالة المؤشر (أربع مراحل من الاختبار إلى العلاج).

**النتائج:**

الأشخاص الذين يعانون من ارتفاع ضغط الدم أو مرض السكري، بالإضافة إلى حالات الأيض القلبي الأخرى كانوا أكثر عرضة للتقدم من خلال سلسلة الرعاية لحالة المؤشر، عن هؤلاء الذين لا يعانون من حالات الأيض القلبي (نسبة المخاطر: 1.14، فاصل ثقة 95٪: 1.09 إلى 1.20، ونسبة مخاطر: 2.18، فاصل الثقة 95%: 1.52 إلى 5.17، على التوالي). تم الربط بين وجود المراضة المشتركة المتعارضة، والتقدم الأكبر في الرعاية لأولئك الذين يعانون من ارتفاع ضغط الدم ولكن ليس مرض السكري. بالنسبة للمصابين بعدوى فيروس نقص المناعة البشرية (HIV) بالإضافة إلى حالات الأيض القلبي، فقد شهدوا تقدماً أقل في مراحل الرعاية مقارنة مع غير المصابين بهذه الحالات (نسبة المخاطر: 0.86، فاصل الثقة 95%: 0.80 إلى 0.92 ).

**الاستنتاج:**

كان المرضى الذين يعانون من حالات متطابقة أكثر احتمالا للتقدم أكثر على طول سلسلة الرعاية، في حين أن أولئك الذين لديهم حالات تعدد مراضة متعارضة، كانوا أكثر ميلاً لعدم التقدم بعد التشخيص. ****

## 摘要

**目的:**

旨在探讨南非农村地区高血压、糖尿病和人类免疫缺陷病毒 (HIV) 感染的老年人中，多重病症如何影响他们在连续护理过程中的进展。

**方法:**

我们分析了 4447 名年龄在 40 岁或以上人群的数据，他们参与了位于阿金库尔分区的纵向研究。于 2014 年 11 月至 2015 年 11 月间完成了家庭访谈。对于高血压和糖尿病患者（分别为 2813 人和 512 人），我们将协调关系定义为其他心脏代谢疾病，将不协调关系定义为精神错乱或人类免疫缺陷病毒 (HIV) 感染。对于人类免疫缺陷病毒 (HIV) 感染（1027 人)，我们将任何其他条件定义为不协调关系。回归模型适用于评估多重病症类型、护理连续体阶段的进展以及患者每个阶段（从测试到治疗的四个阶段）指数状态的可能性）之间的关系。

**结果:**

患有高血压或糖尿病以及其他心脏代谢疾病的患者比那些患有高血压或糖尿病而没有心脏代谢疾病的患者更易在护理连续体阶段的指数状态下取得进展（相对危险度，RR：1.14，95% 置信区间，CI：1.09–1.20，和 RR：2.18，95％ 置信区间，CI：分别为 1.52–3.26)。处于不协调关系的并存病对高血压患者的护理进展有更大的影响，对糖尿病患者无太大影响。人类免疫缺陷病毒 (HIV) 感染并患有心脏代谢疾病的患者与无此症状的患者相比在治疗阶段进展较慢 (RR：0.86，95％ 置信区间，CI：0.80-0.92）。

**结论:**

处于协调关系的患者更有可能在护理连续体阶段取得进展，而处于不协调关系的多重病症患者往往无法在确诊后取得进展。

## Резюме

**Цель:**

Изучение влияния наличия нескольких заболеваний на ход оказания медицинской помощи пожилым пациентам, страдающим гипертензией, диабетом и инфицированных вирусом иммунодефицита человека (ВИЧ), которые проживают в сельских районах Южной Африки.

**Методы:**

Авторы проанализировали данные 4447 пациентов в возрасте старше 40 лет, которые участвовали в лонгитюдном исследовании в субрайоне Аджинкорт. Опросы семейств проводились с ноября 2014 года по ноябрь 2015 года. Для гипертензии и диабета (2813 и 512 человек соответственно) были определены конкордантные состояния (такие как другие кардиометаболические расстройства) и дискордантные состояния (такие как психические расстройства или наличие ВИЧ-инфекции). При наличии ВИЧ-инфекции (1027 человек) все остальные заболевания определялись как дискордантные. Были построены регрессионные модели для оценки взаимозависимости между тем, заболевания какого типа выступают как сопутствующие, и ходом оказания медицинской помощи, а также для определения вероятности того, что пациент находится на той или иной стадии оказания помощи для индексного заболевания (четыре стадии — от тестирования до лечения).

**Результаты:**

Пациенты с гипертензией или диабетом при наличии других кардиометаболических расстройств имели большую вероятность быстрого получения медицинской помощи по индексному заболеванию, чем пациенты без кардиометаболических расстройств (относительный риск, ОР: 1,14; 95%-й доверительный интервал, ДИ: 1,09–1,20 и ОР: 2,18; 95%-й ДИ: 1,52–3,26 соответственно). Наличие дискордантных сопутствующих заболеваний ассоциировалось с более быстрым получением медицинской помощи в случае гипертензии, но не в случае диабета. Пациенты с ВИЧ и кардиометаболическими расстройствами отставали в получении помощи по стадиям ухода в сравнении с пациентами, не имеющими таких расстройств (ОР: 0,86; 95%-й ДИ: 0,80–0,92).

**Вывод:**

Пациенты с конкордантными расстройствами имели большую вероятность быстрого получения медицинской помощи, а пациенты с наличием нескольких дискордантных заболеваний обычно не продвигались далее постановки диагноза.

## Introduction

Increases in ageing populations in low- and middle-income countries has contributed to a rising prevalence of multimorbidity, commonly defined as persons with more than one medical condition.^1^ Previous studies have found that multimorbidity is associated with poorer clinical outcomes,^2^ higher health expenditure and frequency of service use,^3^^–^^6^ higher use of secondary than primary care,^7^^,^^8^ and higher hospitalization rates among patients.^3^^,^^6^^,^^9^

One limitation in the existing literature is that studies of multimorbidity often focus on simple counts of medical conditions. However, different combinations of diseases may affect a person’s health and health care differently. To account for these differences, disease combinations can be categorized as either concordant (similar in risk profile and management) or discordant (not directly related in pathogenesis or management).^10^ Theoretically, concordant conditions are more likely to be diagnosed and treated along with the index condition, because clinical guidelines often incorporate their interactions. For discordant conditions, however, the competing demands of dealing with different conditions may affect the quality of care provided.^11^ Previous studies in high-income settings found that patients with diabetes^12^^,^^13^ or hypertension^14^^,^^15^ had higher odds of achieving testing and control goals when they had concordant conditions than discordant conditions. Diabetes patients with discordant conditions, on the other hand, had higher unplanned use of hospital services and specialized care than those with concordant conditions.^16^

Little is known about the care of patients with human immunodeficiency virus (HIV) and multimorbidity, although studies in the United States of America found that patients with HIV received poorer care for their coexisting conditions than did those without HIV.^17^^–^^19^ Much less is known about how the type of multimorbidity (concordant or discordant) affects a person’s progression along the continuum of care in low- and middle-income countries. Our study aimed to fill this gap by studying the progression along the care continuum among people in South Africa with hypertension, diabetes or HIV infection, all prominent conditions contributing to the complex health transition underway in the country. Furthermore, this study assessed the effect of the type of multimorbidity on HIV care (and not on non-HIV comorbidities) among patients infected with HIV. 

## Methods

### Study design

We analysed cross-sectional data from patients enrolled in the Health and Aging in Africa: a Longitudinal Study of an INDEPTH Community in South Africa. The main study is based the sub-district of Agincourt, in the Bushbuckridge area of Mpumalanga province in South Africa.^20^ The study enrolled 5059 participants aged 40 years and older. Household-based interviews were completed between November 2014 and November 2015 using a primary survey instrument to collect data about respondents’ demographic profile, medical conditions and economic status. More details on data collection are described elsewhere.^21^

The study received ethical approvals from the University of the Witwatersrand human research ethics committee, the Mpumalanga province research and ethics committee, and the Harvard T.H. Chan School of Public Health office of human research administration.

### Study setting

The Agincourt sub-district has six clinics and two health centres, and there are three district hospitals located 25–60 km from the study site.^20^^,^^22^ Primary health-care services are free of charge and most of out-of-pocket health expenditure for patients is incurred for transport, caregiver costs or private health care.

The Integrated Chronic Disease Management model was recently introduced in South Africa to address several elements of managing multimorbidity, including standardized clinical care based on national treatment protocols, and promotion of disease monitoring and management among patients.^23^^–^^25^ In Agincourt, a patient with any symptom or disease arriving at a local clinic will be received by a nurse who is expected to address all the patient’s needs. Those who visit the clinic primarily for HIV testing are directed to a nearby building staffed by health workers tasked solely with HIV testing. Patients are referred for the same management as other patients only if they are diagnosed as HIV positive. 

### Definitions

For this analysis, we studied three index conditions: (i) hypertension; (ii) diabetes; and (iii) HIV infection. We defined an index condition as a reference condition for which the continuum of care was evaluated, not as the time sequence in occurrence or diagnosis of multiple conditions.^26^ For example, for an individual with hypertension plus other conditions, we assigned hypertension as the index condition and evaluated progression along the continuum of care for hypertension in relation to the presence of different types of either concordant or discordant multimorbidity. In addition to the three index conditions, we selected five others as concordant or discordant conditions: (i) dyslipidaemia; (ii) angina; (iii) depression; (iv) post-traumatic stress disorder; and (v) alcohol dependence. We ascertained the presence of the medical conditions based on the clinical diagnosis or clear clinical criteria (Box 1). We selected the medical conditions according to the data that were available in the main study, described in detail elsewhere.^31^

Box 1 **Definitions of conditions in the study of multimorbidity and the care continuum in Agincourt sub-district, South Africa**

**Index conditions**

Hypertension was defined as either mean systolic blood pressure ≥ 140 mmHg and mean diastolic blood pressure ≥ 90 mmHg or patients’ self-report of receiving current treatment.

Diabetes was defined as fasting blood glucose ≥ 126 mg/dL (defined as patients whose last meal was > 8 hours before specimen collection), non-fasting blood glucose ≥ 200 mg/dL or self-reported current treatment.

Human immunodeficiency virus (HIV) status was ascertained either from collected dried blood spots that showed HIV infection or exposure to antiretroviral therapy or self-reported disease status.

**Concordant and discordant conditions**

Dyslipidaemia was one of the following criteria: self-reported disease status; elevated total cholesterol (≥ 6.21 mmol/L); low high-density lipoprotein cholesterol (1.19 mmol/L); elevated low-density lipoprotein cholesterol (> 4.10 mmol/L); elevated triglycerides (> 2.25 mmol/L).

Angina was diagnosed using the Rose chest pain questionnaire.^27^

Depression was defined as three or more symptoms of depression on the Center for Epidemiological Studies depression scale 8-item questionnaire.^28^

Post-traumatic stress disorder was diagnosed as four or more symptoms on a seven-symptom screening scale.^29^

Alcohol dependence was defined using the CAGE questionnaire.^30^

We determined concordance and discordance based on the risk factors and multimorbidities for diagnosis and treatment in the South African national guidelines for hypertension and diabetes.^32^^–^^34^ We found no definition of concordant diseases beyond opportunistic infections in the national HIV guidelines. For people with hypertension, we categorized other cardiometabolic conditions (dyslipidaemia, diabetes and angina) as concordant conditions, and mental disorders (depression, post-traumatic stress disorder and alcohol dependence) and HIV infection as discordant. Similarly, for people with diabetes, we classified other cardiometabolic conditions (hypertension, dyslipidaemia and angina) as concordant conditions, and mental disorders and HIV infection as discordant. For people with HIV, we considered any of the other conditions as discordant.

We defined the continuum of care for each index condition by four sequential stages of care for a patient: being tested for the disease (stage 1), knowing his or her diagnosis (stage 2), ever being initiated on treatment (stage 3) and currently being retained on treatment (stage 4). For hypertension and diabetes, the stage reached was determined from a patient’s self-reporting. For HIV, we relied on both self-reported status and blood test results to determine progression. Patients with dried blood-spot results that showed exposure to antiretroviral therapy (ART) were considered to have reached the treatment stage and all preceding stages, even if they self-reported otherwise.

### Statistical analyses

We first conducted descriptive analyses of the prevalence of the three index conditions as well as the prevalence of concordant and discordant conditions by key sociodemographic covariates. Next, we constructed a count variable for each index condition to signify how many stages each respondent with that index condition had advanced along the corresponding continuum of care for that index condition, with a minimum count of zero and maximum of four.

We fitted quasi-Poisson regression models to analyse the relationship between the number of stages respondents reached in the continuum of care and the type of multimorbidity. We used a series of logistic regression models to estimate the odds ratio (OR) and 95% confidence interval (CI) for associations between either concordant or discordant multimorbidities and the odds of advancing to each stage of the care continuum, conditional on having reached the previous stage. In the case of diagnosis, the logistic regression modelled the unconditional odds. We adjusted all regression models for sociodemographic covariates, including age, sex, education, country of origin, marital status, household size, employment status, having limitations in activities of daily living and wealth (measured in quintiles based on household asset ownership) and synthesized these using standard methods.^35^

All analyses were conducted in R software version 3.3.1 (R Foundation for Statistical Computing, Vienna, Austria).

## Results

Complete data on disease prevalence and continuum of care were available for 4447 respondents (88% of the whole sample of 5059). We excluded 135 people due to missing data about disease status of at least one disease category and 477 people due to missing dried blood-spot samples. 

Table 1 shows the prevalence of hypertension (63%, 2813 people), diabetes (12%, 512 people) and HIV (23%, 1027 people) as well as the prevalence of concordant and discordant conditions by sociodemographic covariates. Among patients with hypertension, 1535 (55%) had one or more additional cardiometabolic condition, 615 (22%) had one or more mental disorder and 480 (17%) were HIV positive. Among those with diabetes, 465 (91%) patients had other cardiometabolic conditions, 139 (27%) had mental disorders and 77 (15%) were HIV positive. Among patients with HIV infection, 728 (71%) presented with cardiometabolic conditions and 181 (18%) with mental disorders. Reflecting the wider population profile, people with HIV tended to be younger, poorer, in employment and separated from partners compared with those with hypertension and diabetes.

**Table 1:** Prevalence of concordant and discordant multimorbidity and sociodemographic profile of patients with hypertension, diabetes and HIV infection in Agincourt sub-district, South Africa, November 2014 to November 2015

Variable	Index condition, no. (%) of people		
	Hypertension	Diabetes	HIV infection
**Total**	2813 (100)	512 (100)	1027 (100)
**Other conditions**			
Cardiometabolic conditions^a^ (excluding index condition)	1535 (55)	465 (91)	728 (71)
Mental disorders^b^	615 (22)	139 (27)	181 (18)
HIV infection	480 (17)	77 (15)	NA
**Age group, years**			
40–49	353 (13)	45 (9)	306 (30)
50–59	757 (27)	125 (24)	382 (37)
60–69	801 (28)	165 (32)	237 (23)
70–79	554 (20)	116 (23)	89 (9)
80+	348 (12)	61 (12)	13 (1)
**Sex**			
Male	1194 (42)	214 (42)	472 (46)
Female	1619 (58)	298 (58)	555 (54)
**Education**			
**No formal education**	1333 (47)	217 (42)	419 (41)
Some primary education (1–7 years)	987 (35)	208 (41)	360 (35)
Some secondary education (8–11 years)	294 (10)	46 (9)	160 (16)
Completed secondary (12+ years)	199 (7)	41 (8)	88 (9)
**Country of origin**			
South Africa	1998 (71)	408 (80)	672 (65)
Mozambique or other	815 (29)	104 (20)	355 (35)
**Marital status**			
Never married	96 (3)	19 (4)	75 (7)
Currently married or living with partner	1457 (52)	269 (53)	409 (40)
Separated or divorced	350 (12)	54 (11)	207 (20)
Widowed	910 (32)	170 (33)	336 (33)
**Household size**			
Living alone	281 (10)	49 (10)	152 (15)
Living with 1 other person	297 (11)	57 (11)	107 (10)
Living in 3–6 people household	1348 (48)	245 (48)	481 (47)
Living in 7+ people household	887 (32)	161 (31)	287 (28)
**Employment status**			
Employed part- or full-time	397 (14)	61 (12)	220 (21)
Other	2416 (86)	451 (88)	807 (79)
**Has limitations in activities of daily living**			
No	2558 (91)	444 (87)	964 (94)
Yes	255 (9)	68 (13)	63 (6)
**Wealth index**			
Quintile 1 (poorest)	527 (19)	62 (12)	253 (25)
Quintile 2	545 (19)	84 (16)	206 (20)
Quintile 3	542 (19)	105 (21)	213 (21)
Quintile 4	600 (21)	121 (24)	195 (19)
Quintile 5 (richest)	599 (21)	140 (27)	160 (16)

CI: confidence interval; HIV: human immunodeficiency virus; NA not applicable.

^a^ Dyslipidaemia, angina, hypertension, diabetes.

^b^ Depression, post-traumatic stress disorder, alcohol dependence.

Notes: These data are based on a total of 4447 people who were tested for the index conditions during the household interview. Inconsistencies arise in some values due to rounding.

### Continuum of care

**Fig. 1:**
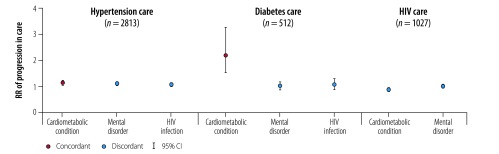
Association between concordant and discordant multimorbidity and progression in the care continuum for patients in Agincourt sub-district, South Africa, November 2014 to November 2015

CI: confidence interval; HIV: human immunodeficiency virus; RR: relative risk.

Notes: Coefficients from the Poisson regression models are expressed as RRs of progression in the care continuum. Covariates included in the model: age group, sex, education, country of origin, household size, marital status, employment status, having limitations in activities of daily living and wealth quintile. Progression in the care continuum is expressed as number of stages reached, minimum = 0, maximum = 4. Stage 1: tested; 2: know status; 3 ever treated; 4: currently treated.

Table 2 shows the number of patients reaching each stage of care for each index condition by sociodemographic covariates. The mean number of stages reached in the care continuum (maximum 4) were 2.44 (standard deviation, SD: 1.50) for hypertension, 2.29 (SD: 1.67) for diabetes and 2.99 (SD: 1.54) for HIV infection. People with hypertension or diabetes plus other cardiometabolic (i.e. concordant) conditions were more likely to proceed further along the care continuum for the index condition than those without cardiometabolic conditions (relative risk, RR: 1.14; 95% CI: 1.09–1.20 and RR: 2.18; 95% CI: 1.52–3.26 respectively; Table 3; Fig. 1). Patients with hypertension and discordant conditions were also more likely to progress further in hypertension care (RR: 1.10; 95% CI: 1.04–1.16 for mental disorders and RR: 1.08; 95% CI: 1.01–1.15 for HIV infection), but those with diabetes were not. Other covariates that were associated with the progression of care among people with hypertension included being older or female, having limitations in activities of daily living, higher education level, of South African origin and wealthier. For those with HIV infection, having cardiometabolic (i.e. discordant) conditions were associated with less advanced progression in HIV care compared with people without cardiometabolic conditions (RR: 0.86; 95% CI: 0.80–0.92). Other covariates that were associated with the further progression of care included being older, male and living in larger households.

**Table 2:** Progression through stages in the care continuum, by multimorbidity status and key sociodemographic covariates, among patients with hypertension, diabetes and HIV infection in Agincourt sub-district, South Africa, November 2014 to November 2015

Variable	Index condition by stage of care reached, no. of patients															
	Hypertension						Diabetes					HIV infection				
	Tested (all patients)^a^		Tested (among those with condition)^b^	Know status^b^	Ever treated^b^	Currently treated^b^	Tested (all patients)^a^	Tested (among those with condition)^b^	Know status^b^	Ever treated^b^	Currently treated^b^	Tested (all patients)^a^	Tested (among those with condition)^b^	Know status^b^	Ever treated^b^	Currently treated^b^
**Total observations**		4447	2813	2084	1915	1508	4447	512	383	300	252	4447	1027	913	730	703
**Reached stage**		3116	2084	1915	1508	1115	2138	383	300	252	224	2993	913	730	703	699
**Other conditions**																
Cardiometabolic conditions (excluding index condition)		1600	1252	1122	906	683	1782	369	288	245	219	2361	648	520	495	491
Mental disorders		694	503	493	403	301	470	112	83	68	64	595	163	134	130	129
HIV infection		717	360	326	239	176	473	52	43	37	30	913	913	730	703	699
**Age group, years**																
40–49		506	229	183	107	71	337	27	19	15	15	613	262	205	191	189
50–59		847	546	471	348	247	574	93	75	68	62	946	355	285	276	275
60–69		831	615	576	464	356	590	119	95	74	72	793	216	170	167	166
70–79		582	430	429	363	271	403	96	75	63	49	437	69	62	62	62
80+		350	264	256	226	170	234	48	36	32	26	204	11	8	7	7
**Sex**																
Male		1361	804	682	512	378	1213	225	170	144	131	1651	486	387	367	364
Female		1755	1280	1233	996	737	925	158	130	108	93	1342	427	343	336	335
**Education**																
No formal education		1454	1002	940	780	578	930	165	128	105	90	1193	365	288	279	277
Some primary (1–7 years of education)		1097	744	695	537	396	785	152	114	100	93	1127	327	267	260	258
Some secondary (8–11 years of education)		349	211	167	120	91	254	32	27	22	20	395	142	111	108	108
Secondary or more (12+ years of education)		216	127	113	71	50	169	34	31	25	21	278	79	64	56	56
**Country of origin**																
South Africa		2174	1491	1404	1112	833	1548	305	241	203	184	2125	601	489	469	467
Mozambique or other		942	593	511	396	282	590	78	59	49	40	868	312	241	234	232
**Marital status**																
Never married		152	61	55	38	28	80	11	11	8	7	159	64	48	45	45
Currently married or living with partner		1591	1069	949	723	531	1120	200	156	132	118	1600	363	293	281	281
Separated or divorced		388	245	214	173	124	276	41	30	24	20	405	191	150	146	145
Widowed		985	709	697	574	432	662	131	103	88	79	829	295	239	231	228
**Household size**																
Living alone		316	187	174	142	106	199	37	29	23	19	295	133	99	97	97
Living with another person		345	234	212	169	120	229	47	37	28	27	331	97	78	73	72
Living with 3–6 persons		1486	977	908	708	525	1040	180	136	120	107	1455	437	355	343	340
Living with 7+ persons		969	686	621	489	364	670	119	98	81	71	912	246	198	190	190
**Employment status**																
Employed part- or full-time		467	265	210	138	97	339	44	36	28	23	554	193	149	142	142
Other		2649	1819	1705	1370	1018	1799	339	264	224	201	2439	720	581	561	557
**Has limitations in activities of daily living **																
No		2779	1858	1698	1304	968	1904	325	249	212	189	2753	849	681	656	652
Yes		337	226	217	204	147	234	58	51	40	35	240	64	49	47	47
**Wealth quintile **																
Quintile 1 (poorest)		613	374	324	251	175	382	45	35	28	26	567	214	171	163	161
Quintile 2		613	403	354	284	203	390	58	45	41	37	578	190	146	142	141
Quintile 3		637	414	380	287	217	425	75	56	47	44	588	186	144	140	140
Quintile 4		610	435	404	329	252	450	95	71	59	48	610	174	149	142	141
Quintile 5 (richest)		643	458	453	357	268	491	110	93	77	69	650	149	121	116	116

^a^ This column shows the number of people among the entire sample were tested for the disease by a provider (regardless of whether they had the index condition).

^b^ This column shows the numbers of people with the index condition who reached this care stage.

**Table 3:** Relative risk for progression through stages in the care continuum, by covariates, among patients with hypertension, diabetes and HIV infection in Agincourt sub-district, South Africa, November 2014 to November 2015

Variable	Index condition		
	Hypertension	Diabetes	HIV infection
**Total no. of people**	2813	512	1027
**Mean no. (SD) of stages reached in the care continuum^a^**	2.44 (1.50)	2.29 (1.67)	2.99 (1.54)
**RR (95% CI) for progression in care**			
Other conditions			
Cardiometabolic conditions^b^ (excluding index condition)	1.14 (1.09–1.20)^d^	2.18 (1.52–3.26)^d^	0.86 (0.80–0.92)^e^
Mental disorders^c^	1.10 (1.04–1.16)^e^	1.02 (0.88–1.19)^e^	0.99 (0.91–1.08)^e^
HIV infection	1.08 (1.01–1.15)^e^	1.08 (0.89–1.31)^e^	NA
**Age group, years**			
40–49	Ref.	Ref.	Ref.
50–59	1.17 (1.07–1.29)	1.26 (0.94–1.71)	1.10 (1.01–1.21)
60–69	1.30 (1.18–1.43)	1.23 (0.91–1.68)	1.07 (0.96–1.19)
70–79	1.42 (1.28–1.57)	1.27 (0.92–1.77)	1.04 (0.90–1.20)
80+	1.36 (1.21–1.52)	1.21 (0.84–1.75)	1.00 (0.72–1.37)
**Sex**			
Male	Ref.	Ref.	Ref.
Female	1.24 (1.17–1.31)	0.99 (0.85–1.16)	0.92 (0.85–0.99)
**Education**			
No formal education	Ref.	Ref.	Ref.
Some primary (1–7 years of education)	0.98 (0.93–1.04)	1.00 (0.85–1.18)	1.06 (0.97–1.16)
Some secondary (8–11 years of education)	0.90 (0.82–0.99)	1.11 (0.84–1.46)	1.04 (0.92–1.17)
Secondary or more (12+ years of education)	0.86 (0.76–0.97)	1.19 (0.88–1.58)	1.01 (0.87–1.18)
**Country of origin**			
South Africa	Ref.	Ref.	Ref.
Mozambique or other	0.92 (0.87–0.98)	0.95 (0.79–1.14)	0.98 (0.91–1.07)
**Marital status**			
Never married	0.96 (0.83–1.11)	0.95 (0.62–1.40)	0.94 (0.81–1.08)
Currently married or living with partner	Ref.	Ref.	Ref.
Separated or divorced	0.93 (0.86–1.01)	0.93 (0.72–1.19)	1.06 (0.97–1.17)
Widowed	0.95 (0.90–1.01)	1.02 (0.86–1.22)	1.05 (0.96–1.14)
**Household size**			
Living alone	Ref.	Ref.	Ref.
Living with another person	1.06 (0.96–1.18)	1.02 (0.76–1.37)	1.09 (0.95–1.25)
Living with 3–6 people	1.02 (0.93–1.11)	0.99 (0.77–1.28)	1.17 (1.05–1.30)
Living with 7+ people	1.03 (0.94–1.13)	1.01 (0.78–1.33)	1.06 (0.95–1.20)
**Employment status**			
Employed part- or full-time	0.95 (0.88–1.03)	0.98 (0.77–1.23)	0.98 (0.90–1.07)
Other	Ref.	Ref.	Ref.
**Has limitations in activities of daily living**			
No	Ref.	Ref.	Ref.
Yes	1.12 (1.04–1.21)	1.23 (1.01–1.48)	1.11 (0.97–1.26)
**Wealth quintile**			
Quintile 1 (poorest)	Ref.	Ref.	Ref.
Quintile 2	1.04 (0.96–1.12)	0.95 (0.74–1.24)	1.05 (0.95–1.16)
Quintile 3	1.08 (1.00–1.17)	0.96 (0.75–1.23)	1.01 (0.91–1.12)
Quintile 4	1.09 (1.01–1.18)	0.97 (0.77–1.25)	1.05 (0.94–1.17)
Quintile 5 (richest)	1.21 (1.11–1.31)	1.02 (0.80–1.32)	1.07 (0.95–1.21)
**Constant**	1.46 (1.26–1.69)	0.85 (0.48–1.48)	2.78 (2.35–3.28)

CI: confidence interval; NA: not applicable; Ref.: reference group; RR: relative risk; SD: standard deviation.

^a^ Minimum = 0, maximum = 4. Stage 1: tested; 2: know status; 3 ever treated; 4: currently treated.

^b^ Dyslipidaemia, angina, hypertension, diabetes.

^c^ Depression, post-traumatic stress disorder, alcohol dependence.

^d^ Concordant.

^e^ Discordant.

Note: Dependent variable was progression in the care continuum (number of stages reached by each patient).

### Stages of care reached

#### Hypertension

**Fig. 2:**
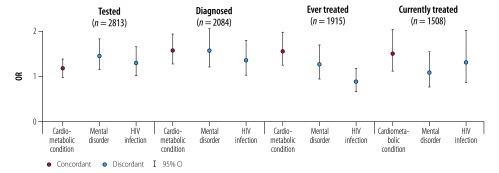
Association between concordant and discordant multimorbidity and progression in the continuum of hypertension care for patients with hypertension in Agincourt sub-district, South Africa, November 2014 to November 2015

HIV: human immunodeficiency virus; OR: odds ratio.

Notes: Coefficients from the logistic regression models are expressed as ORs. Covariates included in the model: age group, sex, education, country of origin, household size, marital status, employment status, having limitations in activities of daily living and wealth quintile.

Looking more closely at each stage of the continuum, having discordant medical conditions was associated with a higher likelihood of being tested for hypertension. This was true both among the entire sample (OR: 1.32; 95% CI: 1.11–1.57 for patients with mental disorders; OR: 1.20; 95% CI: 1.02–1.42 for those with HIV infection) and those with hypertension (OR: 1.44; 95% CI: 1.15–1.82 with mental disorders; OR: 1.29; 95% CI: 1.01–1.65 with HIV infection; Table 4; Fig. 2). Having mental disorders was also associated with a higher likelihood of being diagnosed with hypertension (OR: 1.52; 95% CI: 1.17–1.99), but was not associated with any of the remaining stages in the care continuum. Having HIV infection was not associated with progress in any stages of care among people with hypertension. In comparison, patients with one or more cardiometabolic (concordant) conditions were more likely to be diagnosed with hypertension (OR: 1.53; 95% CI: 1.24–1.88), ever-treated (OR: 1.52; 95% CI: 1.21–1.92) and currently on treatment (OR: 1.46; 95% CI: 1.08–1.97) for hypertension.

**Table 4:** Odds of progression through stages in the care continuum for patients with hypertension, diabetes and HIV infection and concordant or discordant multimorbidity in Agincourt sub-district, South Africa, November 2014 to November 2015

Index condition	Stage of care reached				
	Tested (all patients)	Tested (among those with condition)	Know status (among those tested)	Ever treated (among those who know status)	Currently treated (among those ever treated)
**Hypertension**					
No. of observations	4447	2813	2084	1915	1508
aOR (95% CI) of associations with:					
Cardiometabolic conditions^a^	1.13 (0.99–1.29)	1.17 (0.98–1.39)	1.53 (1.24–1.88)	1.52 (1.21–1.92)	1.46 (1.08–1.97)
Mental disorders^b^	1.32 (1.11–1.57)	1.44 (1.15–1.82)	1.52 (1.17–1.99)	1.22 (0.91–1.64)	1.04 (0.74–1.50)
HIV infection^b^	1.20 (1.02–1.42)	1.29 (1.01–1.65)	1.31 (0.99–1.74)	0.85 (0.63–1.14)	1.26 (0.83–1.95)
**Diabetes**					
No. of observations	4447	512	383	300	252
aOR (95% CI) of associations with:					
Cardiometabolic conditions^a^	1.75 (1.51–2.04)	4.20 (2.19–8.19)	3.55 (1.34–9.64)	3.03 (0.67–12.21)	2.88 (0.27–22.57)
Mental disorders^b^	1.13 (0.97–1.31)	1.36 (0.82–2.31)	0.76 (0.44–1.33)	0.72 (0.35–1.55)	1.68 (0.57–5.50)
HIV infection^b^	1.10 (0.94–1.28)	1.07 (0.60–1.98)	1.29 (0.62–2.87)	0.81 (0.32–2.18)	0.43 (0.14–1.40)
**HIV infection**					
No. of observations	4447	1027	913	730	703
aOR (95% CI) of associations with:					
Cardiometabolic conditions^b^	1.06 (0.90–1.25)	1.03 (0.66–1.58)	0.46 (0.30–0.69)	0.32 (0.09–0.87)	0.00 (NA)
Mental disorders^b^	0.99 (0.85–1.17)	1.03 (0.61–1.83)	0.98 (0.64–1.53)	1.20 (0.41–4.44)	0.57 (0.06–12.26)

aOR: adjusted odds ratio; CI: confidence interval; HIV: human immunodeficiency virus; NA: not applicable.

^a^ Concordant conditions.

^b^ Discordant conditions.

Notes: For hypertension, concordant conditions were other cardiometabolic conditions (dyslipidaemia, diabetes, angina); discordant conditions were mental disorders (depression, post-traumatic stress disorder, alcohol dependence) and HIV infection. For diabetes, concordant conditions were other cardiometabolic conditions (hypertension, dyslipidaemia, angina); discordant conditions were mental disorders (depression, post-traumatic stress disorder, alcohol dependence) and HIV infection. For HIV infection, there were no concordant conditions; discordant conditions were cardiometabolic conditions (hypertension, diabetes, dyslipidaemia and angina) and mental disorders (depression, post-traumatic stress disorder, alcohol dependence). Covariates included in the model: age group, sex, education, country of origin, household size, marital status, employment status, having limitations in activities of daily living, and wealth quintile.

#### Diabetes

**Fig. 3:**
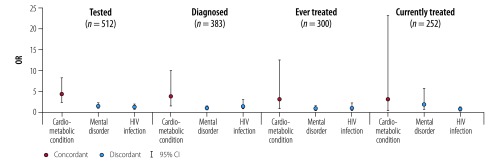
Association between concordant and discordant multimorbidity and progression in the continuum of diabetes care for patients with diabetes in Agincourt sub-district, South Africa, November 2014 to November 2015

HIV: human immunodeficiency virus; OR: odds ratio.

Notes: Coefficients from the logistic regression models are expressed as ORs. Covariates included in the model: age group, sex, education, country of origin, household size, marital status, employment status, having limitations in activities of daily living and wealth quintile.

The effects of the types of multimorbidity on people with diabetes were greater. Having cardiometabolic (concordant) conditions was associated with higher odds of being tested for diabetes both among the whole sample (OR: 1.75; 95% CI: 1.51–2.04) and those with diabetes (OR: 4.20; 95% CI: 2.19–8.19; Table 4; Fig. 3). Among patients with diabetes, having cardiometabolic conditions was associated with higher odds of knowing their diabetes status (OR: 3.55; 95% CI: 1.34–9.64), but not of being initiated or retained on treatment. Having discordant conditions (mental disorder or HIV infection) was not associated with progression to each stage. 

#### HIV infection

**Fig. 4:**
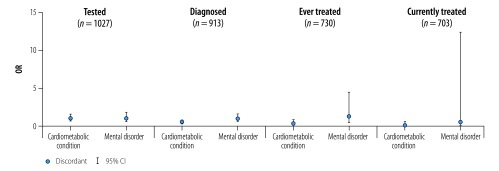
Association between concordant and discordant multimorbidity and progression in the continuum of HIV care for patients with HIV infection in Agincourt sub-district, South Africa, November 2014 to November 2015

HIV: human immunodeficiency virus; OR: odds ratio.

Notes: Coefficients from the logistic regression models are expressed ORs. Covariates included in the model: age group, sex, education, country of origin, household size, marital status, employment status, having limitations in activities of daily living and wealth quintile.

In contrast with hypertension and diabetes, having HIV and cardiometabolic (discordant) conditions was associated with worse care for HIV patients. The odds were 54% lower for knowing their HIV status (OR: 0.46; 95% CI: 0.30–0.69) and 68% lower for ever receiving ART (OR: 0.32; 95% CI: 0.09–0.87; Table 4; Fig. 4). 

Data for the adjusted odds ratios for each covariate by stage of care reached are available from the corresponding author.

## Discussion

In line with theories and empirical findings from high-income settings,^12^^–^^14^ we found that having concordant conditions was associated with a higher likelihood of progressing further along the continuum of care for hypertension and diabetes in our study population. This may be explained by the emphasis that the South African hypertension guidelines place on diabetes and dyslipidaemia as important comorbidities, and the emphasis on hypertension and dyslipidaemia in the diabetes guidelines.^32^^,^^33^ These guidelines do not give much emphasis to HIV, although both mention it, and neither mention mental disorders. Moreover, providers may be more inclined to treat concordant conditions urgently to reach the target treatment outcomes for the index condition. For example, treating dyslipidaemia in patients may lead to targeting blood pressure control, because of the benefits of preventing the progression of coronary artery diseases.^36^

On the other hand, having discordant conditions was not associated with worse care progression for hypertension and diabetes, contrary to experience in high-income settings.^11^^,^^13^ Although some studies have shown that mental disorders are associated with poorer progression in care for cardiometabolic conditions,^36^ we did not find a significant effect. Negative findings were observed only among people with HIV, where the presence of cardiometabolic (discordant) conditions was associated with less progress in HIV care. This is a concerning finding given that both HIV infection and the use of ART have been associated with increased risk of coronary heart disease and myocardial infarction.^37^^,^^38^ Previous studies found lower quality of care for non-HIV conditions among HIV patients.^17^^–^^19^ Factors that may have contributed to those findings include the lack of specific guidelines for HIV patients for treating diseases other than opportunistic infections; prioritization of short-term health needs; and the difficulty of balancing the demands of caring for complex patients with other medical and psychosocial problems.

Comparing across each stage in the continuum of care, both hypertension and diabetes patients with concordant or discordant conditions had a higher likelihood of reaching the first stages of care. This may be due to the lower opportunity costs involved for health-care providers and patients in relation to testing and diagnosis, versus those related to initiation and adherence to treatment. Testing and diagnosing hypertension involve simple procedures with relatively little effort required by providers, and thus the presence of any type of multimorbidity may increase the chance that the patient will be tested. However, the positive effect of discordant diseases may recede as the opportunity cost increases, as is the case for being initiated on and supported to adhere to treatment. More effort is required on the part of the practitioner to determine the right regimen, initiate the treatment, provide counselling on adherence and follow-up regularly to ensure the desired outcomes are met.

Patients who have non-diabetes cardiometabolic conditions may be tested for diabetes, given the overlap in the risk factors, pathophysiological pathways and treatment guidelines. We did not see this positive effect of multimorbidity among people who were HIV-infected, perhaps due to stigma, practitioners’ lower awareness of HIV among older people and the fact HIV testing requires more complex laboratory-based assessment than measuring blood pressure. Furthermore, we suggest that the negative association between HIV care and having cardiometabolic diseases may relate in part to how the clinics in Agincourt are organized. The separate procedure for HIV testing may explain why people with only HIV and no other conditions were more likely to be diagnosed with HIV conditional on being tested since they likely entered the clinic solely for receiving HIV care.

The findings also imply that the objective of the South Africa’s Integrated Chronic Disease Management model may not yet be realized. While not examined empirically in our study, barriers such as long waiting times, staff shortages and drug stock-outs may have negatively impacted the implementation of the management model and resulted in fewer visits made by the patients and shorter consultation times with providers.^25^ The nurses may not be trained to diagnose or manage all diseases, and, given time constraints, they are often only able to address the patient's chief complaint and, in some cases, the concordant diseases that are listed in the guidelines.^25^ For nationwide implementation of the integrated chronic disease management model, our findings suggest the need for improvements in leveraging one programme (such as the HIV programme) for scaling-up services for another condition (such as noncommunicable disease services), for example by putting more effort into ensuring patient engagement in stages with higher opportunity costs. There may be potential for benefits through the introduction of programmes, such as the Sustainable East Africa Research in Community Health’s campaign and the United States President’s Emergency Plan for AIDS Relief.^39^^–^^41^ Implementing such joint programmes would make cardiometabolic disease management available alongside HIV services to bring populations with different types of multimorbidity into care.

Our study is subject to several limitations. First, we assessed whether the presence of a concordant or discordant condition was associated with progression in the care continuum, not whether being in care for one disease leads to being in care for another. Due to the cross-sectional nature of this study, we could not determine the time sequencing of the conditions or the care progression. We were also unable to assess causality on which type of multimorbidity affects care progression. Second, while the prevalence of the three index conditions and the concordant and discordant conditions were based on clinical criteria, data on the stages to which people progressed were self-reported, and our results therefore may have over- or underestimated coverage of different services. As the excluded samples were most commonly due to missing HIV measurements (due to patients’ refusal to be tested), it is likely that we have underestimated the prevalence of HIV infection. The HIV prevalence within the sample is similar to the prevalence level found earlier in Agincourt.^42^ Third, all conditions within the cardiometabolic and mental conditions were weighted equally, whereas it is plausible that specific combinations of diseases are associated with higher likelihood of progressing further along the care continuum. Finally, the study’s comparability with existing studies and generalizability to settings with low HIV prevalence may be limited.

We conclude that the presence of any type of multimorbidity is associated with a higher likelihood of being in stages of care with lower opportunity costs, while the presence of concordant conditions is associated with higher likelihood of being in stages with higher opportunity costs. Our findings from a relatively typical setting in rural South Africa have policy implications for enhancing access to testing and treatment services to improve service coverage and population health in the country. While we could not corroborate causality, further research, informed by forthcoming waves of the main study, will improve our understanding of the impact of different types of multimorbidity on health outcomes and the use of health services.
